# Beyond Ventricular Enlargement: Multimodal MRI Assessment Improves Surgical Decision-Making in Normal Pressure Hydrocephalus

**DOI:** 10.3390/brainsci16060654

**Published:** 2026-06-20

**Authors:** Duygu Baykal, Mustafa Umut Etli, Muhammed Enes Atik, Zekeriya Gedikli, Mehmet Ziya Çetiner, Ahmet Kürşat Kara, Semra Işık

**Affiliations:** 1 Department of Neurosurgery, Bursa City Hospital, Bursa 16250, Türkiye; 2 Department of Neurosurgery, Antalya City Hospital, Antalya 07080, Türkiye; 3 Department of Neurosurgery, Ümraniye Training and Research Hospital, İstanbul 34764, Türkiye

**Keywords:** normal pressure hydrocephalus, morphometry, disproportionately enlarged subarachnoid space hydrocephalus, high-convexity tightness, ventriculoperitoneal shunt

## Abstract

**Highlights:**

**What are the main findings?**

- Conventional ventricular indices such as the Evans index, FOHR, and bicaudate index were insufficient to distinguish surgical candidates among patients with suspected normal pressure hydrocephalus.
- High-convexity tightness emerged as the only independent MRI predictor associated with shunt surgery, while combined imaging models demonstrated the highest diagnostic accuracy.

**What are the implications of the main findings?**

- Morphological MRI features reflecting CSF dynamics may provide greater clinical value than ventricular size measurements alone in surgical decision-making for NPH.
- Integrating multiple imaging parameters with clinical assessment may improve the identification of patients who are most likely to benefit from shunt surgery.

**Abstract:**

Objectives: This study aimed to evaluate the utility of MRI-based morphometric and qualitative parameters in identifying patients with suspected normal pressure hydrocephalus (NPH) associated with shunt surgery selection following clinical and lumbar puncture evaluation. Methods: We retrospectively analyzed 134 participants: 84 symptomatic patients evaluated for suspected NPH and 50 age-matched controls with normal brain MRI findings. Symptomatic patients were categorized according to subsequent clinical management following lumbar puncture evaluation into those who underwent shunt surgery (Shunt group) and those who received conservative management (Conservative group). The Evans index, fronto-occipital horn ratio (FOHR), bicaudate index, callosal angle, ventricular measurements, and disproportionately enlarged subarachnoid space hydrocephalus (DESH) components were analyzed. The discriminatory performance of MRI parameters for shunt surgery selection was assessed using ROC analysis; independent predictors of shunt surgery selection were determined using logistic regression. Results: Although conventional ventricular indices and ventricular dimensions were significantly greater in symptomatic patients than in the control group (*p* < 0.001), baseline continuous MRI measurements did not significantly differ between the Shunt and Conservative groups (*p* > 0.05). Callosal angle demonstrated no discriminatory value for shunt surgery selection. In univariate analyses, an Evans Index > 0.36, a bicaudate index > 0.23, and a DESH score > 2 were associated with shunt surgery selection. High-convexity tightness and an Evans Index > 0.36 differed significantly between groups and remained independently associated with shunt surgery selection in multivariable analysis. Conclusions: Ventricular width-based indices alone appear insufficient for identifying patients selected for shunt surgery among individuals evaluated for suspected NPH. Both qualitative and quantitative MRI features, particularly high-convexity tightness and an Evans Index > 0.36, were independently associated with shunt surgery selection following routine clinical assessment. Integrating multimodal imaging parameters with clinical evaluation may provide a more reliable approach for identifying patients who are ultimately selected for shunt surgery following lumbar puncture assessment.

## 1. Introduction

Normal pressure hydrocephalus (NPH) is a potentially reversible neurological disorder characterized by gait disturbance, cognitive decline, and urinary incontinence, particularly affecting older adults [1]. Although this classical triad is well known, the diagnosis of NPH remains challenging because of significant overlap with other neurodegenerative and cerebrovascular disorders and the absence of a definitive diagnostic test. Consequently, identifying patients who are most likely to be considered for shunt surgery in routine clinical practice remains a major clinical challenge.

Magnetic resonance imaging (MRI) plays a central role in the evaluation of patients with suspected NPH and provides important diagnostic information beyond ventricular enlargement alone [2]. Commonly assessed imaging parameters include the Evans index, fronto-occipital horn ratio (FOHR), bicaudate index, and callosal angle. In addition, the disproportionately enlarged subarachnoid space hydrocephalus (DESH) pattern has emerged as an important supportive imaging finding [3,4,5]. Although these markers are useful in describing the radiological profile of NPH, their clinical significance may vary among patients.

In clinical practice, imaging findings are interpreted in conjunction with symptom severity, neurological examination findings, comorbidities, and the expected benefit of shunt surgery. Accordingly, some patients with prominent ventriculomegaly may be managed conservatively, whereas others with less pronounced imaging abnormalities may be considered for surgery because of a more compelling overall clinical presentation [2,6,7,8]. Therefore, reliance on a single radiological threshold may not adequately reflect real-world surgical decision-making in suspected NPH.

Most previous studies have evaluated these MRI parameters individually, with limited direct comparison of multiple morphometric and qualitative imaging features across clinically distinct patient groups [9,10,11,12]. In this study, we compared multiple MRI-based morphometric and qualitative parameters among surgically treated patients with suspected NPH, conservatively managed symptomatic patients, and controls with normal brain MRI findings. Our aim was to determine whether these markers differ between groups and whether they are associated with shunt surgery selection following clinical and lumbar puncture assessment in patients with suspected NPH.

## 2. Materials and Methods

This retrospective study enrolled symptomatic patients assessed for suspected NPH at the Neurosurgery Clinics of Ümraniye Training and Research Hospital and Bursa City Hospital between January 2020 and January 2026, as well as age-matched controls with normal brain MRI findings.

Initially, 110 symptomatic patients and 50 controls were evaluated. Among the patients in the symptomatic group, 26 were excluded because of missing clinical or radiological data or the presence of secondary hydrocephalus. The final analysis included 134 participants, comprising 84 symptomatic patients and 50 controls.

The study population was divided into three groups: (1) patients who underwent ventriculoperitoneal shunt surgery (Shunt group), (2) patients managed conservatively after lumbar puncture (LP) (Conservative group), and (3) age-matched controls without hydrocephalus or intracranial abnormalities on MRI.

Inclusion criteria for the symptomatic groups included clinical findings consistent with the Adams–Hakim triad, including gait disturbance, cognitive impairment, and/or urinary incontinence, and the availability of pre-treatment MRI data. The control group comprised individuals without clinical or radiological findings of hydrocephalus or other intracranial pathology. All symptomatic patients had undergone neurological evaluation before referral to the neurosurgery clinic. Patients considered by the neurology team to have alternative neurological disorders as the primary explanation for their symptoms were not referred for neurosurgical shunt evaluation.

All symptomatic patients (*n* = 84) underwent high-volume lumbar puncture (30–50 mL CSF drainage) for diagnostic evaluation. Following the procedure, patients were clinically reevaluated for changes in gait, cognitive function, and urinary symptoms. Because of the retrospective nature of the study and the real-world clinical setting, no predefined quantitative threshold or standardized scoring system was uniformly applied. Decisions regarding shunt surgery were based on the overall clinical assessment documented after lumbar puncture and the multidisciplinary evaluation process. Patients considered to have improved following lumbar puncture were offered ventriculoperitoneal shunt surgery, whereas those without apparent clinical improvement were managed conservatively. The timing of post-lumbar puncture evaluation was based on routine clinical practice and was not standardized across all patients because of the retrospective nature of the study.

MRI images were retrospectively evaluated. Radiological measurements were performed independently by two researchers, one observer from each center, without knowledge of the clinical data and treatment groups. Inter-rater reliability was formally assessed for all radiological parameters. For continuous measurements, values obtained by the two observers were averaged following reliability confirmation. For qualitative and semiquantitative variables, disagreements were resolved through consensus to establish the final dataset used for subsequent analyses.

The evaluated radiological parameters included the FOHR, Evans index, bicaudate index, callosal angle, temporal horn width, third ventricle width, and fourth ventricle diameter. Findings related to disproportionately enlarged subarachnoid space hydrocephalus (DESH) were also assessed using a semiquantitative scoring system. The components characterizing DESH—Sylvian fissure enlargement, high-convexity tightness, and periventricular CSF leakage—were graded as 0 (absent), 1 (mild), 2 (moderate), and 3 (marked), and the total DESH score was used as a continuous variable in the analyses.

All measurements were obtained from standard axial and coronal sections using digital imaging software (OsiriX MD v13.0 Pixmeo SARL, Bernex, Switzerland).

Demographic and clinical data, including presenting symptoms, symptom duration, treatment modality, and post-treatment clinical response, were retrieved from patient records. Clinical improvement after LP and changes in gait, cognitive functions, and urinary symptoms were determined according to physician assessment.

### Statistical Analysis

Descriptive statistics are presented as median (minimum–maximum) values for continuous variables and as frequencies with percentages for categorical variables. Data normality was assessed using the Shapiro–Wilk test.

Inter-rater reliability between the two independent observers was formally evaluated for all radiological metrics prior to data aggregation. For continuous morphometric measurements, the Intraclass Correlation Coefficient (ICC) was calculated using a two-way mixed-effects model with absolute agreement. Following reliability confirmation, measurements obtained by the two observers were averaged for subsequent analyses. For qualitative and semiquantitative radiological markers, interobserver agreement was assessed using Cohen’s kappa statistics. Any discrepancies were subsequently resolved by consensus to establish the final dataset used for analysis.

Group comparisons were performed using the Kruskal–Wallis test with Bonferroni-adjusted pairwise comparisons for continuous variables. Categorical variables were analyzed using the Pearson chi-square test or Fisher–Freeman–Halton test, as appropriate.

Effect sizes were calculated using epsilon-squared (ε^2^) for Kruskal–Wallis analyses. The strength of categorical associations was evaluated using Phi (ɸ) and Cramér’s V.

The discriminatory performance of MRI parameters in identifying patients selected for shunt surgery was assessed using receiver operating characteristic (ROC) curve analysis, with the area under the curve (AUC) reported along with 95% confidence intervals. Optimal cut-off values were identified using the Youden Index, and corresponding sensitivity, specificity, positive predictive value (PPV), and negative predictive value (NPV) were calculated. ROC curves were compared using the DeLong test.

Binary logistic regression analysis was performed to identify independent predictive variables for shunt surgery. Variables demonstrating statistical significance (*p* < 0.05) in univariate analysis were included in the multivariable model. Prior to multivariable modeling, correlations and multicollinearity diagnostics were evaluated among candidate predictors. Model fit was assessed employing the Omnibus and Hosmer–Lemeshow tests, and explained variance was reported as Nagelkerke’s R^2^. Internal validation was conducted through bootstrap resampling (1000 iterations).

All statistical analyses were conducted using the IBM SPSS Statistics software (version 26.0) (IBM Corp., Armonk, NY, USA). A *p*-value < 0.05 was considered statistically significant.

## 3. Results

### Inter-Rater Reliability Analysis

Prior to group comparisons, inter-rater reliability analyses were performed across the total cohort to confirm the consistency of radiological assessments between the two independent observers. Quantitative morphometric measurements demonstrated excellent reproducibility. The Intraclass Correlation Coefficient (ICC) was 0.81 (95% CI: 0.72–0.87; *p* < 0.001) for the Evans Index, 0.81 (95% CI: 0.73–0.86; *p* < 0.001) for the FOHR, 0.998 (95% CI: 0.996–0.999; *p* < 0.001) for the bicaudate index, 0.88 (95% CI: 0.84–0.91; *p* < 0.001) for the callosal angle, and 0.91 (95% CI: 0.87–0.93; *p* < 0.001) for the mean temporal horn width. For qualitative and semiquantitative markers, Cohen’s kappa statistics demonstrated excellent agreement, with a kappa value of 1.00 (*p* < 0.001) for high-convexity crowding, total DESH score, Sylvian fissure dilation, and periventricular CSF seepage in the finalized assessment dataset.

The demographic and baseline clinical characteristics of the study population (*n* = 134) are summarized in Table 1. The median age of the cohort was 66 years and did not differ significantly among groups (*p* = 0.271). Sex distribution showed a significant intergroup difference (*p* = 0.031), corresponding to a small-to-moderate association (Cramér’s V = 0.227). The proportion of male patients was higher in the Shunt group than in the control group (62% vs. 36%; *p* < 0.05). The Conservative group exhibited an intermediate distribution, with no significant differences relative to either group (*p* > 0.05).

Clinical symptom profiles were evaluated in symptomatic patients only (*n* = 84). Gait disturbance was highly prevalent and similarly distributed in the Shunt and Conservative groups (96% vs. 97%; *p* = 1.000). Cognitive impairment was more prevalent in patients who received shunt treatment than in those managed conservatively (82% vs. 53%; *p* = 0.004), demonstrating a moderate association (φ = 0.312). Similarly, urinary incontinence was more common among shunt-treated patients than those managed conservatively (88% vs. 68%; *p* = 0.023), reflecting a small-to-moderate association (φ = 0.249). Median symptom duration was 18 months in the Shunt group and 12 months in the Conservative group, without a statistically significant intergroup difference (*p* = 0.166). Preoperative LP was performed in most patients in both groups (90% in the Shunt group and 100% in the Conservative group), with no significant difference observed in preoperative evaluation practices (*p =* 0.078).

Comparative analysis of morphometric MRI parameters revealed significant differences among the three study groups (Table 2). The Evans Index (ε^2^ = 0.58), FOHR (ε^2^ = 0.70), and bicaudate index (ε^2^ = 0.69) all differed significantly among groups (all *p* < 0.001), indicating large between-group effect sizes. Pairwise comparisons demonstrated significantly higher values in both the Shunt and Conservative groups than in the control group (all adjusted *p* < 0.001). However, no significant differences were observed between the Shunt and Conservative groups for the Evans Index (adjusted *p* = 0.471), FOHR (adjusted *p* = 1.000), or bicaudate index (adjusted *p* = 0.324).

Callosal angle measurements differed significantly among the three groups (*p* < 0.001, ε^2^ = 0.49), indicating a large between-group effect size. Both the Shunt group (88.03° [35.20–128.18]) and the Conservative group (81.53° [38.05–129.65]) demonstrated narrower callosal angles compared with the control group (122.92° [110.24–135.79]; both adjusted *p* < 0.001). However, no significant difference was noted between the Shunt and Conservative groups (adjusted *p* = 1.000). Consistent with this finding, ROC analysis showed that the callosal angle had no significant discriminatory value for shunt surgery selection (AUC = 0.56, 95% CI: 0.45–0.67; *p* = 0.384).

Ventricular size measurements were consistent with these findings. Temporal horn width (ε^2^ = 0.58), third ventricle width (ε^2^ = 0.63), and fourth ventricle diameter (ε^2^ = 0.44) differed significantly across the three groups (all *p* < 0.001), reflecting large effect sizes. Pairwise analyses demonstrated significantly greater ventricular dimensions in both the Shunt and Conservative groups compared with the control group (all adjusted *p* < 0.001). However, no significant differences were observed between the Shunt and Conservative groups for temporal horn width (adjusted *p* = 1.000), third ventricle width (adjusted *p* = 0.835), or fourth ventricle diameter (adjusted *p* = 0.127).

**Figure 1:**
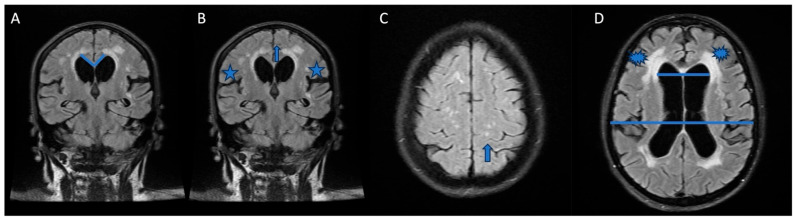
Representative MRI findings evaluated in the present study: (**A**) Coronal FLAIR image demonstrating Evans Index measurement. (**B**) Coronal FLAIR image illustrating the DESH pattern, including Sylvian fissure enlargement (blue star-shaped markers) and high-convexity tightness (arrow). (**C**) Axial FLAIR image demonstrating high-convexity tightness with narrowing of the superior convexity sulci (arrow). (**D**) Axial FLAIR image showing ventriculomegaly with Evans Index measurement and associated periventricular CSF seepage (asterisks).

The distribution of qualitative MRI markers across the study groups is presented in Table 3. The prevalence of the DESH pattern differed significantly across groups (*p* < 0.001; Cramér’s V = 0.623). Pairwise comparisons revealed that DESH was more common in the Shunt and Conservative groups than in the control group (100% and 91% vs. 42%; *p* < 0.05 for both), whereas no difference was observed between the two symptomatic cohorts. Representative MRI examples of the evaluated qualitative imaging features are shown in Figure 1.

Sylvian fissure dilation exhibited a significant overall difference among groups (*p* = 0.033; Cramér’s V = 0.226). Pairwise analyses revealed a higher proportion in the Shunt group than in the control group (68% vs. 42%; *p* < 0.05), whereas the Conservative group (56%) showed an intermediate distribution without statistically significant differences relative to either group (*p* > 0.05).

High-convexity tightness also varied across the three groups (*p* < 0.001; Cramér’s V = 0.59), with a higher prevalence in the Shunt group than in the Conservative group (60% vs. 18%; *p* < 0.05). Both symptomatic groups demonstrated higher proportions than the control group (*p* < 0.05 for both comparisons).

Periventricular CSF seepage also differed across groups (*p* = 0.001; Cramér’s V = 0.332). The proportion was higher in both the Shunt and Conservative groups compared with the controls (64% and 65% vs. 30%; *p* < 0.05 for both), with no difference between the two symptomatic cohorts.

**Figure 2:**
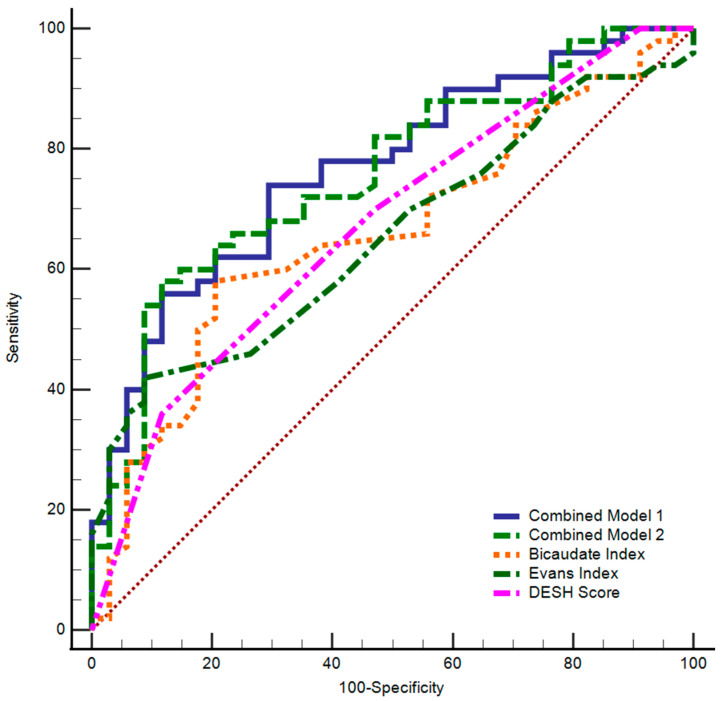
ROC curves of the standalone MRI parameters and combined multivariable models for identifying shunt candidates. The red dashed diagonal line represents the line of no discrimination (AUC = 0.5), indicating the performance expected by random chance.

To identify MRI-derived measures associated with shunt surgery selection, ROC analyses were performed (Table 4, Figure 2).

The DESH score demonstrated discriminatory performance (AUC = 0.67, 95% CI: 0.56–0.77; *p* = 0.002) in the symptomatic cohorts (*n* = 84). Using the optimal threshold of >2, the DESH score yielded a specificity of 88.2% and a PPV of 81.8%.

The Evans Index demonstrated statistically significant discriminatory performance (AUC = 0.65, 95% CI: 0.54–0.76; *p* = 0.009). At a cut-off value of >0.36, it achieved a specificity of 91.2% and a PPV of 87.5%. The bicaudate index also demonstrated significant discriminatory value (AUC = 0.66, 95% CI: 0.55–0.76; *p* = 0.009). Conversely, FOHR (AUC = 0.55, 95% CI: 0.43–0.65; *p* = 0.477) and callosal angle (AUC = 0.56, 95% CI: 0.45–0.67; *p* = 0.384) did not demonstrate significant standalone discriminatory value.

Combined Model 1, integrating the Evans Index, callosal angle, and DESH score, achieved a highly significant AUC of 0.77 (95% CI: 0.66–0.85; *p* < 0.001), with 74.0% sensitivity and 70.6% specificity. Combined Model 2, incorporating the Evans Index, bicaudate index, and DESH score, demonstrated comparable performance (AUC = 0.76, 95% CI: 0.65–0.84; *p* < 0.001), with a remarkably high specificity of 88.2% and a PPV of 87.9%. To compare the discriminatory performance of the two combined models, the DeLong comparison showed no significant difference between them (difference between areas = 0.01, 95% CI: −0.05 to 0.07; *p* = 0.737).

Univariate and multivariable logistic regression analyses were performed to identify variables associated with shunt surgery selection (Table 5). In univariate analyses, age and sex were not significantly associated with shunt surgery selection. Among quantitative MRI indices, an Evans Index > 0.36 was associated with higher odds of shunt surgery selection (crude OR 7.48, 95% CI 2.02–27.77; *p* = 0.003), as was a bicaudate index > 0.23 (crude OR 2.87, 95% CI 1.17–7.07; *p* = 0.022). FOHR and callosal angle were not significantly associated with the outcome. Among ventricular dimensions, the fourth ventricle diameter demonstrated a significant univariate association (crude OR 1.03, 95% CI 1.00–1.05; *p* = 0.030), whereas the mean temporal horn width and the third ventricle width did not. Among qualitative MRI markers, a DESH score > 2 was associated with higher odds of shunt surgery selection (crude OR 4.22, 95% CI 1.28–13.90; *p* = 0.018), and high-convexity crowding showed a strong univariate association (crude OR 7.00, 95% CI 2.46–19.96; *p* < 0.001).

Multivariable Model Statistics: Multivariable statistics were derived from the final step (Step 4) of an automated backward stepwise (Conditional) logistic regression framework, following an initial univariable screening, filtering variables at *p* < 0.05. The backward elimination process systematically removed the DESH score threshold at Step 2, the fourth ventricle diameter at Step 3, and the bicaudate index threshold at Step 4 to satisfy the stringent Events Per Variable (EPV) criterion based on the 34 limiting non-shunt events and to prevent model overfitting. Multivariable Model Statistics (Finalized Step 4): Omnibus Test of Model Coefficients: χ^2^(2) = 23.70, *p* < 0.001; Hosmer–Lemeshow Test: χ^2^(2) = 1.43, *p* = 0.490; Nagelkerke R^2^ = 0.332; Overall Classification Accuracy = 72.6%.

Collinearity metrics (highest VIF = 1.63, maximum Condition Index = 26.92) confirmed the model’s absolute stability and the absence of severe multicollinearity.

OR: odds ratio; CI: confidence interval. Statistically significant values (*p* < 0.05) are highlighted in bold.

Variables significant in univariate analyses were entered into a multivariable logistic regression model using a backward stepwise selection approach. During model refinement, the DESH score threshold, fourth ventricle diameter, and bicaudate index threshold were removed sequentially. The final multivariable model demonstrated satisfactory fit (Omnibus χ^2^(2) = 23.70, *p* < 0.001; Hosmer–Lemeshow *p* = 0.490) and explained 33.2% of the variance (Nagelkerke R^2^ = 0.332), with an overall classification accuracy of 72.6%.

In the final adjusted model, an Evans Index > 0.36 and high-convexity crowding remained independently associated with shunt surgery selection. Patients with an Evans Index > 0.36 had higher odds of being selected for shunt surgery than those below this threshold (adjusted OR 5.98, 95% CI 1.51–23.68; *p* = 0.011). Similarly, high-convexity crowding was independently associated with shunt surgery selection (adjusted OR 5.91, 95% CI 1.98–17.61; *p* = 0.001). Collinearity diagnostics demonstrated no evidence of severe multicollinearity, with the highest VIF value of 1.63 and a maximum Condition Index of 26.92.

## 4. Discussion

NPH continues to be a clinically important entity in neurosurgical practice as a potentially reversible cause of cognitive decline, gait disturbance, and urinary dysfunction. In this setting, radiological parameters are increasingly being utilized not only to support diagnosis but also to inform clinical decision-making in patients being evaluated for shunt surgery.

In this study, a comprehensive set of morphometric and qualitative MRI parameters was systematically analyzed by comparing patients with suspected NPH who underwent surgical treatment, those managed conservatively, and controls with normal MRI findings. The main finding was that conventional ventricular size-based measures clearly distinguished symptomatic patients from controls but had limited value in separating patients ultimately selected for shunt surgery from those managed conservatively. In contrast, an Evans Index > 0.36 and high-convexity tightness remained independently associated with shunt surgery selection after multivariable adjustment.

Ventricular enlargement is traditionally considered a hallmark of NPH, with the Evans index widely used in both clinical and research settings [13,14,15]. A key finding of our study is that values for conventional ventricular indices, including the Evans index, FOHR, and bicaudate index, while significantly elevated compared with controls, did not differ significantly between the surgically treated and conservatively managed patients. This indicates that, although ventricular enlargement is a characteristic feature of NPH, it does not serve as a standalone discriminative marker for surgical decision-making.

Although the callosal angle has been reported to be useful in differentiating NPH from other etiologies of ventriculomegaly, it did not show significant discriminatory performance in distinguishing surgical candidates in our study [16,17,18,19,20]. Both symptomatic groups had narrower callosal angles than controls, but no significant difference was observed between the Shunt and Conservative groups. This suggests that the callosal angle may help characterize the broader NPH imaging phenotype but may not independently distinguish patients selected for shunt surgery in a clinically preselected symptomatic population.

In the multivariable analysis, both an Evans Index > 0.36 and high-convexity tightness remained independently associated with shunt surgery selection. This finding indicates that ventricular enlargement and qualitative CSF space redistribution may provide complementary information. While ventricular indices quantify global ventricular size, high-convexity tightness reflects narrowing of the superior convexity subarachnoid spaces despite ventricular enlargement. This configuration is a key component of the DESH phenotype and may help distinguish iNPH from ventriculomegaly related to cerebral atrophy, in which cortical sulci are generally enlarged rather than compressed, consistent with previous observations comparing iNPH and hydrocephalus ex vacuo phenotypes [10,19].

High-convexity tightness may therefore capture a morphological feature that is not adequately represented by ventricular width alone. Sylvian fissure enlargement reflects expansion of basal CSF spaces, whereas high-convexity tightness captures the complementary phenomenon of convexity sulcal narrowing. These markers are related within the DESH construct but may not contribute equally to clinical decision-making. In our analysis, the DESH score was associated with shunt surgery selection in univariate analysis but did not retain independent significance after multivariable adjustment. This suggests that the discriminatory contribution of the DESH pattern may not be uniformly distributed across its components and that high-convexity tightness may represent the most clinically informative element of this imaging phenotype in our cohort.

Beyond conventional radiological features, recent studies have explored ventricular morphometric characteristics, glymphatic-related biomarkers, and cerebrospinal fluid dynamics as complementary approaches for patient characterization and outcome prediction. Together, these findings support the concept that multimodal assessment strategies may provide more clinically relevant information than isolated imaging markers alone [5,21,22].

An alternative interpretation of our findings is that high-convexity tightness may not simply represent a marker of surgical selection but rather a feature of a more typical DESH-related iNPH phenotype. Conversely, patients lacking high-convexity tightness may represent a more heterogeneous group, potentially including individuals with atrophy-dominant, neurodegenerative, or cerebrovascular processes despite presenting with NPH-like symptoms. Therefore, the observed association between high-convexity tightness and shunt surgery selection should not be interpreted as direct evidence of postoperative shunt responsiveness or surgical benefit.

Although ventricular indices have long been considered fundamental parameters in the literature, our findings show that they are insufficient when used in isolation and that models incorporating multiple parameters provide better discrimination between patients selected for shunt surgery and those managed conservatively. The fact that the combined model, integrating the Evans index, bicaudate index, FOHR, and DESH score, achieved the highest AUC value further underscores that diagnostic and treatment decision-making in NPH is inherently multidimensional. This observation is consistent with emerging evidence suggesting that no single imaging marker adequately captures the biological and clinical heterogeneity of iNPH and that integrated imaging approaches may better reflect disease complexity [23,24,25].

The similar distribution of many radiological parameters between the Shunt and Conservative groups reflects the complexity of patient selection in suspected NPH. In routine practice, treatment decisions are not based on imaging alone but rather on a combination of symptoms, neurological assessment, comorbidities, lumbar puncture response, and clinician judgment. Our findings support this integrated approach and suggest that quantitative ventricular indices and qualitative morphological features should be interpreted together rather than as isolated thresholds.

Although a significant difference in sex distribution was observed across the overall study population, this difference was primarily driven by the control group, whereas the two symptomatic groups demonstrated comparable sex distributions. Furthermore, sex was not significantly associated with shunt surgery selection in the regression analysis. Nevertheless, the potential influence of sex-related anatomical variation on radiological measurements cannot be completely excluded.

A key strength of our study is that the comparative evaluation of radiological parameters was conducted not only in surgically treated patients but also in conservatively managed patients and controls. This design provides a more clinically relevant assessment of how MRI markers relate to real-world management decisions in suspected NPH. However, the control group consisted of individuals with normal MRI findings rather than disease-control subjects with neurodegenerative disorders that may clinically mimic NPH. Therefore, the present findings should not be interpreted as evidence for differentiating NPH from Alzheimer’s disease, Parkinsonian syndromes, vascular cognitive impairment, or other neurodegenerative conditions. Furthermore, biomarker analyses for Alzheimer’s disease, amyloid PET imaging, APOE genotyping, and standardized assessment of small vessel disease burden were not routinely available; therefore, concomitant neurodegenerative or cerebrovascular pathologies could not be completely excluded.

From a clinical standpoint, our findings suggest that a decision-making approach based on ventricular indices may have limitations. In particular, identifying surgical candidates may be made more reliable by jointly evaluating certain morphological features and multiple parameters, rather than relying solely on ventricular width.

Finally, the fact that the surgical decision-making process in this study was evaluated in conjunction with the clinical response after high-volume LP underscores the necessity to interpret imaging findings not in isolation but in an integrated manner with clinical dynamics. Our findings indicate that, despite the limited performance of individual radiological parameters, the combined evaluation of multiple imaging features provides improved discrimination regarding shunt surgery selection. This supports a multidimensional approach in which clinical and radiological data are integrated for a more reliable identification of surgical candidates in NPH.

Importantly, the outcome evaluated in this study was the clinical decision to proceed with shunt surgery following lumbar puncture assessment rather than postoperative clinical improvement. Therefore, the identified imaging markers should be interpreted as factors associated with surgical selection in routine clinical practice rather than definitive predictors of shunt responsiveness or treatment success. Future prospective studies incorporating standardized postoperative outcome measures are needed to determine the predictive value of these imaging features for surgical response.

This study has several limitations. First, the retrospective design limits control over possible confounding factors and may lead to selection bias. Second, post-lumbar puncture clinical improvement was not assessed using a uniform standardized scoring system across all patients, which may have introduced variability in patient selection for shunt surgery. Third, although interobserver reliability was formally assessed, qualitative parameters such as high-convexity tightness and DESH-related features remain partly observer-dependent. Fourth, the absence of disease-control cohorts and systematic neurodegenerative biomarker assessment limits the ability to exclude alternative or concomitant neurological disorders. Finally, although multivariable modeling and collinearity diagnostics were performed, the modest sample size may still have affected model stability. Therefore, the regression findings should be interpreted cautiously and validated in larger prospective cohorts.

## 5. Conclusions

This study demonstrates that conventional ventricular indices and the callosal angle, although effective in distinguishing symptomatic patients from controls, have limited value in guiding surgical decision-making. In contrast, both high-convexity tightness and an Evans Index > 0.36 were independently associated with shunt surgery selection following routine clinical assessment. Furthermore, when evaluated together with the clinical response after LP, models integrating multiple imaging parameters achieved the strongest association with shunt surgery selection within this cohort, supporting the concept that quantitative and qualitative MRI features provide complementary information. These findings suggest that, in patients with suspected NPH, a multidimensional approach integrating clinical assessment and multimodal imaging features may better reflect real-world surgical decision-making than reliance on any single radiological parameter. The identified imaging features should be interpreted as factors associated with patient selection for shunt surgery rather than direct predictors of postoperative clinical improvement or treatment success.

## Figures and Tables

**Table 1:** Demographic and baseline clinical characteristics of the study groups (*n* = 134).

	Shunt Group (*n* = 50)	Conservative Group (*n* = 34)	Control Group (*n* = 50)	*p*
Age (Years)	67.5 [50–86]	63.0 [52–79]	63.0 [53–90]	0.271
Gender				**0.031**
Female	19(38%)	16(47.1%)	32(64%)	
Male	31 (62%)	18 (52.9%)	18 (36%)	
Symptom Duration (Months)	18.0 [2–72]	12.0 [2–60]	N/A	0.166
Clinical Presentation				
Gait Disturbance	48 (96%)	33 (97.1%)	N/A	1.000
Cognitive Impairment	41 (82%)	18 (52.9%)	N/A	**0.004**
Urinary Incontinence	44 (88%)	23 (67.6%)	N/A	**0.023**
Preoperative Lumbar Puncture	45 (90%)	34 (100%)	N/A	0.078

Note: Data are presented as *n* (%) for categorical variables and median [minimum–maximum] for non-normally distributed continuous variables. *p* < 0.05 is considered statistically significant. Bold values indicate statistically significant results (*p* < 0.05). N/A: Not Applicable.

**Table 2:** Comparison of morphometric MRI parameters across study groups (*n* = 134).

Parameters	Shunt Group (*n* = 50)	Conservative Group (*n* = 34)	Control Group (*n* = 50)	*p*
Evans Index	0.35 [0.21–0.51]	0.34 [0.26–0.42]	0.26 [0.22–0.32]	**<0.001**
FOHR	0.46 [0.37–0.66]	0.47 [0.38–0.54]	0.35 [0.16–0.40]	**<0.001**
Bicaudate Index	0.24 [0.15–0.44]	0.22 [0.13–0.40]	0.12 [0.07–0.18]	**<0.001**
Callosal Angle (°)	88.03 [35.20–128.18]	81.53 [38.05–129.65]	122.92 [110.24–135.79]	**<0.001**
Temporal Horn Width (mm)	8.80 [4.10–21.13]	9.79 [3.07–14.32]	4.14 [1.50–8.53]	**<0.001**
3rd Ventricle Width (mm)	16.09 [7.35–30.92]	17.89 [6.90–26.21]	8.07 [2.14–12.09]	**<0.001**
**4th Ventricle Diameter (mm)**	17.79 [11.68–39.71]	15.96 [11.65–27.69]	13.10 [10.26–16.77]	**<0.001**

Note: Data are presented as median [minimum–maximum] for non-normally distributed variables. *p* < 0.05 is considered statistically significant. Bold values indicate statistically significant results (*p* < 0.05).

**Table 3:** Qualitative radiological markers across study groups (*n* = 134).

Qualitative Markers	Shunt Group (*n* = 50)	Conservative Group (*n* = 34)	Control Group (*n* = 50)	*p*
DESH Pattern	50 (100%)	31 (91%)	21 (42%)	**<0.001**
Sylvian Fissure Dilation	34 (68%)	19 (56%)	21 (42%)	**0.033**
High-Convexity Crowding	30 (60%)	6 (18%)	0 (0%)	**<0.001**
**Periventricular CSF Seepage**	32 (64%)	22 (65%)	15 (30%)	**0.001**

Note: Data are presented as *n* (%) for categorical variables. *p* < 0.05 is considered statistically significant. Bold values indicate statistically significant results (*p* < 0.05).

**Table 4:** Association of MRI parameters with shunt surgery selection.

Parameters	AUC (95% CI)	Cut-Off	Sens (%)	Spec (%)	PPV (%)	NPV (%)	*p*
Bicaudate Index	0.66 (0.55–0.76)	>0.23	58.0	79.4	80.6	56.2	**0.009**
FOHR	0.55 (0.43–0.65)	≤0.47	58.0	58.8	67.4	48.8	0.477
Evans Index	0.65 (0.54–0.76)	>0.36	42.0	91.2	87.5	51.7	**0.009**
Callosal Angle	0.56 (0.45–0.67)	>71.35°	84.0	41.2	67.7	63.6	0.384
DESH Score	0.67 (0.56–0.77)	>2	36.0	88.2	81.8	48.4	**0.002**
COMBINED MODEL_I	0.77 (0.66–0.85)	>0.55	74.0	70.6	78.7	64.9	**<0.001**
COMBINED MODEL_II	0.76 (0.65–0.84)	>0.69	58.0	88.2	87.9	58.8	**<0.001**

**Note:** AUC: area under the curve; CI: confidence interval; Sens: sensitivity; Spec: specificity; PPV: positive predictive value; NPV: negative predictive value. All evaluation steps were executed within the finalized symptomatic dataset (*n* = 84) derived from consensus multi-rater measurements. *p* < 0.05 is considered statistically significant. Youden Index J values: Bicaudate Index = 0.37; FOHR = 0.17; Evans Index = 0.33; Callosal Angle = 0.25; DESH Score = 0.24; Combined Model 1 = 0.45; Combined Model 2 = 0.46. Combined Model 1 (*n* = 84): Integrated model based on logistic regression, including Evans Index, Callosal Angle, and DESH score. Combined Model 2 (*n* = 84): Optimized multivariable model including Evans Index, Bicaudate Index, and DESH score (excluding non-significant parameters to utilize the most robust independent predictors and maximize model parsimony). *p* < 0.05 is considered statistically significant. Bold values indicate statistically significant results (*p* < 0.05).

**Table 5:** Univariate and multivariable logistic regression analysis of factors identifying patients selected for shunt surgery.

	Univariate Model		Multivariable Model	
	Crude OR (95% CI)	*p*	Adjusted OR (95% CI)	*p*
Demographics				
Age	1.04 (0.98–1.10)	0.181	-	-
Gender (Male)	1.45 (0.60–3.51)	0.409	-	-
Quantitative MRI Indices				
Evans Index (0.36)	7.48 (2.02–27.77)	**0.003**	5.98 (1.51–23.68)	**0.011**
FOHR	1.22 (0.01–35.77)	0.961	-	-
Bicaudate Index (>0.23)	2.87 (1.17–7.07)	**0.022**	-	-
Callosal Angle (°)	1.01 (1.00–1.01)	0.066	-	-
Ventricular Diameters				
Mean Temporal Horn Width	1.04 (0.99–1.08)	0.099	-	-
3rd Ventricle Width	1.02 (0.99–1.04)	0.165	-	-
4th Ventricle Diameter	1.03 (1.00–1.05)	**0.030**	-	-
Qualitative MRI Markers				
DESH Score (>2)	4.22 (1.28–13.90)	**0.018**	-	-
Sylvian Fissure Dilation	1.68 (0.68–4.13)	0.260	-	-
High-Convexity Crowding	7.00 (2.46–19.96)	**<0.001**	5.91 (1.98–17.61)	**0.001**

**Reference categories:** Female for gender; “Absence” for qualitative MRI markers (Sylvian fissure dilation and high-convexity crowding). *p* < 0.05 is considered statistically significant. Bold values indicate statistically significant results (*p* < 0.05).

## Data Availability

The data supporting the findings of this study are available from the corresponding author upon reasonable request. The data are not publicly available because of privacy and ethical restrictions.

## Abbreviations

NPH, Normal Pressure Hydrocephalus; MRI, Magnetic Resonance Imaging; LP, Lumbar Puncture; CSF, Cerebrospinal Fluid; FOHR, Fronto-Occipital Horn Ratio; DESH, Disproportionately Enlarged Subarachnoid Space Hydrocephalus; ROC, Receiver Operating Characteristic; AUC, Area Under the Curve; PPV, Positive Predictive Value; NPV, Negative Predictive Value; OR, Odds Ratio; SD, Standard Deviation; ANOVA, Analysis of Variance; SPSS, Statistical Package for the Social Sciences; CI, Confidence Interval; VP Shunt, Ventriculoperitoneal Shunt.
